# A method for non-overlapping identification of human myeloid derived suppressor cells

**DOI:** 10.1186/2051-1426-2-S3-P150

**Published:** 2014-11-06

**Authors:** Michael Gustafson, Yi Lin, Mary Maas, Dennis Gastineau, Allan Dietz

**Affiliations:** 1 Mayo Clinic, Rochester, MN, USA

## 

The function of human myeloid derived suppressor cells (MDSCs) cannot be precisely measured without the proper identification of homogeneous populations. Indeed, many published accounts of human MDSCs are really describing multiple distinct populations confused either by sample processing, poor gating strategies, and/or lack of appropriate cell surface markers. To improve the standardization of the identification of human myeloid derived suppressor cells and other myeloid phenotypes, we developed a method for the identification and analysis of human myeloid populations by the use of several 10-color flow cytometric protocols in combination with novel software analyses. This method utilizes the direct staining of peripheral blood that allows for the quantitation of myeloid cells and the delineation of non-overlapping immunophenotypes. The 10-color protocols, tested in both healthy volunteer controls and cancer patients, allow us to define diverse phenotypes that include mature monocytes, granulocytes, circulating dendritic cells, immature myeloid cells, in addition to MDSCs. We demonstrate that human MDSC subsets fall into three distinct populations: CD14^+^HLA-DR^lo/neg ^monocytes, LIN^-^CD33^+^HLA-DR^- ^cells, and CD15^+ ^granulocytes. We additionally identify CD123 as a marker uniquely expressed on LIN^-^CD33^+^HLA-DR^- ^MDSCs. Our method permits us to measure myeloid and MDSC phenotypes in relation to total mononuclear cells, total myeloid cells, total leukocytes and as cell counts. The data generated from our method will allow for more uniform reporting of myeloid and MDSC phenotypes for biomarker development.

